# Sources of Syphilitic Infection

**Published:** 1874-03

**Authors:** James Nevins Hyde


*AMERICAN JOURNAL OF THE MEDICAL SCIENCES.—January*, 1874:

Sources of Syphilitic Infection.—*By James Nevins Hyde, M.D.*—There would, at first thought, seem to be a reasonable prospect of the total disappearance of syphilis from the face of the earth, if every individual exhibiting its primary lesions could be induced to forego the acts by which its virus is propagated. Are there methods by which the virus of syphilis is transmitted from individual to individual of the human family, different from, and in addition to, those with which we have had occasion to be most familiar?

I. *The ordinary menstrual secretion of a syphilitic female is capable of producing chancre and its consecutive symptoms, under such circumstances as would insure contagion from a primary sore.* Syphilographers and the general profession are now agreed respecting the contagiousness of the blood of a syphilitic subject. It is now also admitted that vaccinal syphilis is due to the admixture of the blood of the vaccinifer with the lymph obtained from vesicles—the lymph itself not having been demonstrated to possess the virulent qualities of the blood.

II. *The secretions of a uterus, which is the seat of secondary syphilitic deposits, are capable of producing chancre and its consecutive symptoms, under such circumstances as would insure contagion from primary sores.* The infectious character of catamenial blood in constitutional syphilis being determined, it is clear that the discharges from a uterus which is itself the seat of visceral syphilis must be in an equal degree virulent. Evidences of this fact are not wanting in the writings of late authorities.

III. *A gonorrhœal discharge, in a syphilitic subject, is capable of producing chancre and its consecutive symptoms, under such circumstances as would insure contagion from primary sores.* Since the day when the doctrine of Hunter, as to the identity of gonorrhœal and chancrous virus, was completely refuted, the two diseases have been almost entirely dissociated. There is some danger, consequently, of losing sight of the fact that they still have a common ground. Ricord was among the first to put forth the statement that "gonorrhœa will not produce syphilitic ulcers, and not be followed by secondary syphilis, unless there be a chancre or syphilitic sore in the urethra." To this should be added, "*unless, also, there be constitutional syphilis resulting from a precedent sore.*"

